# Predicting survival in anaplastic astrocytoma patients in a single-center cohort of 108 patients

**DOI:** 10.1186/s13014-020-01728-8

**Published:** 2020-12-17

**Authors:** Helena C. W. Wahner, Malte Träger, Katja Bender, Leonille Schweizer, Julia Onken, Carolin Senger, Felix Ehret, Volker Budach, David Kaul

**Affiliations:** 1 Department of Radiation Oncology, Charité University Hospital Berlin, Augustenburger Platz 1, 13353 Berlin, Germany; 2 Department of Neuropathology, Charité University Hospital Berlin, Berlin, Germany; 3 German Cancer Consortium (DKTK), Partner Site Berlin, German Cancer Research Center (DKFZ), Heidelberg, Germany; 4 Department of Neurosurgery, Charité University Hospital Berlin, Berlin, Germany

**Keywords:** Score, Anaplastic astrocytoma, Overall survival, Glioma

## Abstract

**Background:**

Current guidelines for the treatment of anaplastic astrocytoma (AA) recommend maximal safe resection followed by radiotherapy and chemotherapy. Despite this multimodal treatment approach, patients have a limited life expectancy. In the present study, we identified variables associated with overall survival (OS) and constructed a model score to predict the OS of patients with AA at the time of their primary diagnosis.

**Methods:**

We retrospectively evaluated 108 patients with newly diagnosed AA. The patient and tumor characteristics were analyzed for their impact on OS. Variables significantly associated with OS on multivariable analysis were included in our score. The final algorithm was based on the 36-month survival rates corresponding to each characteristic.

**Results:**

On univariate analysis, age, Karnofsky performance status, isocitrate dehydrogenase status, and extent of resection were significantly associated with OS. On multivariable analysis all four variables remained significant and were consequently incorporated in the score. The total score ranges from 20 to 33 points. We designated three prognostic groups: A (20–25), B (26–29), and C (30–33 points) with 36-month OS rates of 23%, 71%, and 100%, respectively. The OS rate at 5 years was 8% in group A, 61% in group B and 88% in group C.

**Conclusions:**

Our model score predicts the OS of patients newly diagnosed with AA and distinguishes patients with a poor survival prognosis from those with a greater life expectancy. Independent and prospective validation is needed. The upcoming changes of the WHO classification of brain tumors as well as the practice changing results from the CATNON trial will most likely require adaption of the score.

## Background

Anaplastic astrocytoma (AA) is a diffusely infiltrating, malignant primary brain tumor. An update of the World Health Organization (WHO) classification in 2016 established new diagnostic groups based on histological phenotypes and genotypes, which are linked to unique biological behaviors and treatment responses [1]. WHO grade III tumors are distinguished in case of oligodendroglioma and AA. They differ in their molecular profiles, and patients have a distinct median age at diagnosis and median survival. Oligodendrogliomas typically present with 1p/19q-codeletion combined with IDH-mutation and have the best outcome of all WHO grade III tumors. AA can be further differentiated into subgroups based on isocitrate dehydrogenase (IDH) type 1 and 2 mutation status. The prognosis of IDH-mutant AA is intermediate, whereas IDH-wildtype AA is linked to a poor prognosis, bearing many similarities to glioblastoma (GBM).

As our knowledge of molecular markers has rapidly evolved, studies performed before the 2016 WHO classification update did not distinguish between the separate entities, as we currently do. Key research in the past decade lacked a clear distinction between AA and oligodendroglioma. In the NOA-04 study, molecular subgroup analysis of a mixed cohort of patients with WHO grade III tumors demonstrated associations of IDH mutations, 1p/19q-codeletion, and O^6^-methylguanine-DNA-methyltransferase (MGMT) promoter methylation with better progression-free survival (PFS) and overall survival (OS). Together with a young age, a high initial Karnofsky Performance Status (KPS) and the presence of oligodendroglial histological characteristics are generally the most important factors associated with better outcomes of WHO grade III gliomas [2].

Ideal treatments for the different subgroups as defined by the genotype remain uncertain. Radiotherapy (RT), chemotherapy (CTx), and a combination of both have all proven to be effective measures in primary and secondary treatment for different subgroups of diffuse glioma [2–4]. Current treatment for AA consists of maximal safe resection followed by a combination of temozolomide (TMZ)-based chemotherapy and RT [5]. Studies specifically designed to investigate AA treatments are rare. The current recommendations are partly based on preliminary results of the CATNON trial, which compared the effects of RT alone, RT combined with either concomitant or adjuvant TMZ, or RT with both concomitant and adjuvant TMZ on PFS and OS. The first interim results published in 2017 demonstrated a benefit for the two study groups receiving adjuvant CTx [6]. This finding was rapidly adapted for routine clinical use, as data for evidence-based treatment of this specific diagnosis are sparse. The second interim results from 2019 included the first molecular analysis, which limited the benefit of adjuvant TMZ to IDH-mutant AA. The results also demonstrated that concomitant TMZ did not increase OS in the entire study cohort, though a trend towards benefit was present in IDH-mutant tumors [7].

Prognostic scores for the heterogeneous entity “glioma” are well established, especially for recurrent disease. One of the earliest prognostic scores for re-irradiation of recurring glioma was published by Combs and colleagues in 2013 [8]. It was subsequently improved by Kessel et al., adding further predictive variables to the scoring system [9, 10]. In 2018, Niyazi et al. presented a re-irradiation score to predict post-recurrence survival in patients with glioma [11]. Recently, Straube et al. published a score specifically designed to predict survival in elderly patients with newly diagnosed GBM [12]. These scores all have in common that they are based on cohorts that either consist only (Straube et al.) or mostly (Kessel et al., Niyazi et al.) of patients with GBM or low-grade glioma and GBM (Combs et al.) [8, 9, 11, 12]. AA has been underrepresented in this research, and there is a need for more specific scoring systems to predict survival outcomes. Developing a neurooncological treatment strategy for patients with AA presents clinicians with the challenge of balancing maximally effective treatment with quality of life. A diagnosis-specific score applicable at the time of initial diagnosis can help objectify a patient’s prognosis. In the present study, we aimed to identify variables associated with OS in patients with AA. Our goal was to construct a simple score specifically for this diagnostic subgroup that factored in the information available at the point of primary treatment assessment. To our knowledge, no scores based on a homogeneous cohort of patients with AA have been developed and published to date.

## Methods

This was a single-center, retrospective, observational study. Ethical approval was obtained from the Charité Review Board (EA2/150/20). The patient database of Charité Universitätsmedizin Berlin was searched for patients with AA who had received treatment between January 2010 and January 2020. We researched each patient’s medical record to assess the following eligibility criteria: age ≥ 18 years, primary histopathological diagnosis of a WHO grade III tumor, absence of 1p/19q-codeletion, and primary treatment received at Charité Universitätsmedizin Berlin. Treatment decisions at our center are made by a multidisciplinary tumor board and reflect the individual patient’s wishes. As part of a narrow follow-up schedule, all patients receive consultations and contrast-enhanced magnetic resonance imaging (MRI) controls every 3 months. Overall, 108 eligible cases were included. We then reviewed the respective medical records to retrieve information including basic patient characteristics (sex, age, KPS), histopathology of primary diagnosis (IDH status, MGMT promoter methylation status), primary therapy (extent of resection; RT including dosage, fractionation, and planning target volume; CTx including substance, concomitant, or adjuvant administration), disease progression, secondary therapy, last contact, and death.

Six characteristics were analyzed for their potential association with OS: sex (female vs. male), age at the time of surgery (divided by the median age, < 41 vs. ≥ 41 years), KPS (less than vs. greater than or equal to the median of 90%), IDH status (mutant vs. wildtype), MGMT promoter methylation status (methylated vs. non-methylated), and resection status after primary surgery (biopsy vs. subtotal resection vs. gross total resection). The KPS was determined postoperatively. IDH mutation was determined by immunostaining. If IDH R132H was negative, additional pyrosequencing for IDH1/2 was performed. The extent of resection was defined based on postoperative contrast-enhanced MRI.

Statistical analysis was performed using IBM SPSS Statistics for Mac OS, version 26.0 (IBM Corp., Armonk, NY, USA). A *p* value < 0.05 was considered significant. Univariate analysis (UVA) was performed using the log-rank test. Variables that proved to be significant in UVA were included in a multivariable Cox regression analysis (MVA). We included characteristics that were independent predictors of OS in MVA in our scoring system. To generate a subscore for each variable, the 36-month survival rate for each characteristic was divided by 10. The subscores of all significant parameters were totaled to result in the score for each patient. This methodological approach has been demonstrated in other studies [12, 13].

## Results

An overview of the patient and tumor characteristics most relevant for the construction of a prognostic score is presented in Table 1. Primary treatment modalities varied in the investigated cohort. The majority of patients had undergone either CTx alone or RT with concomitant and adjuvant CTx. Almost all patients who underwent RT received total doses of ≥ 59.2 Gy. Normofractionated (1.8–2.0 Gy single dose per day) and accelerated hyperfractionated (1.6 Gy twice daily) RT were the most common fractionation schemes. One patient was treated in line with the Nordic glioma regimen and received hypofractionated treatment with 34 Gy in 10 fractions of 3.4 Gy [14]. If concomitant or adjuvant CTx was administered in the primary setting, the applied drug was almost exclusively TMZ. The standard concomitant dose was 75 mg/m^2^ daily. Standard adjuvant chemotherapy with TMZ included 150 or 200 mg/m^2^/d TMZ administered on 5 consecutive days as part of a 28-day cycle. Most patients received 12 cycles. If interruption or termination occurred at any point of treatment, we attempted to document the duration and reason. When comparing the different treatment arms of our cohort, patients receiving CTx only (44.4%, n = 49) showed significantly better survival (*p* 0.001) than those who received any kind of combined radiochemotherapy (RCTx, 31.5%, n = 34). This can be explained by substantial differences in the frequency of IDH-wildtype tumors: Of those 34 patients receiving RCTx, 7 (20.6%) had IDH-wildtype tumors. In contrast, there were only 2 (4.2%) IDH-wildtype tumors in the 49 patients strong CTx only group. This distribution is in line with the general recommendation for IDH-wildtype tumors to receive combination therapy.

**Table 1:** Patient and tumor characteristics

	n, median (min–max)	%
**Sex**		
Male	64	59.3
Female	44	40.7
**Age** **(years)**	41 (22–87)	
< 41	53	49.1
≥ 41	55	50.9
**KPS** **(%)**	90 (60–100)	
< 90	25	23.1
≥ 90	69	63.9
Unknown	14	13.0
**IDH**		
Mutant	84	77.8
Wildtype	9	8.3
Unknown	15	13.9
**MGMT**		
Methylated	86	79.6
Non-methylated	19	17.6
Unknown	3	2.8
**1p/19q-Codeletion**		
Non-codeleted	108	100.0
Codeleted	0	0.0
Unknown	0	0.0
**Resection**		
Biopsy	13	12.0
Subtotal resection	25	23.1
Gross total resection	65	60.2
Unknown	5	4.6
**Treatment after surgery**		
CTx mono	48	44.4
RT mono	8	7.4
RT + concCTx	5	4.6
RT + adjCTx	5	4.6
RT + concCTx + adjCTx	20	18.5
RT, CTx unknown	6	5.6
No RT, CTx unknown	3	2.8
Unknown	13	12.0

*KPS* Karnofsky performance status, *IDH* isocitrate dehydrogenase type 1 and 2, *MGMT* O6-methylguanine-DNA-methyltransferase, *CTx* chemotherapy, *RT* radiotherapy, *concCTx* concomitant chemotherapy, *adjCTx* adjuvant chemotherapy

Median follow-up to last contact or death was 29.5 months. Forty patients underwent salvage therapy for recurrent disease. Salvage treatment regimens were quite heterogeneous. Nine patients received trimodal therapy with re-resection followed by RCTx. Eight patients had re-resection followed by either radiotherapy or systemic therapy. Six patients underwent re-resection without adjuvant treatment. Seven patients received RCTx without re-resection. Five patients received systemic monotherapy and another five patients received only radiotherapy without re-resection.

**Fig. 1:**
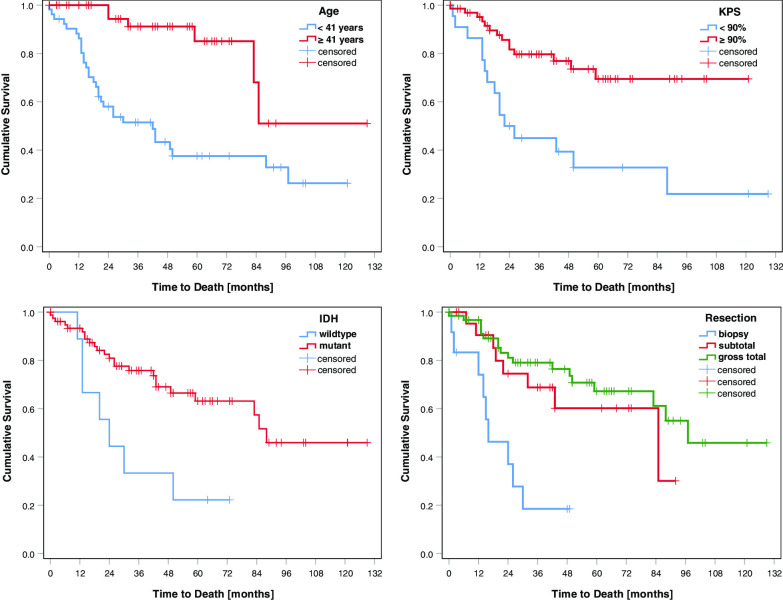
Kaplan–Meier curves for parameters qualifying for inclusion in the score

**Fig. 2:**
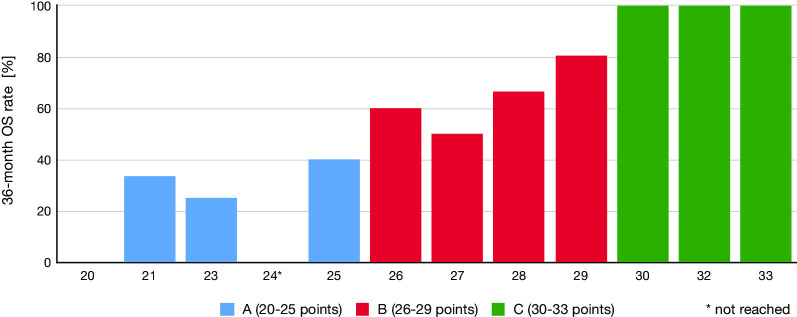
Total score for individual patients and respective 36-month OS rate divided in three prognostic groups. * not reached

**Fig. 3:**
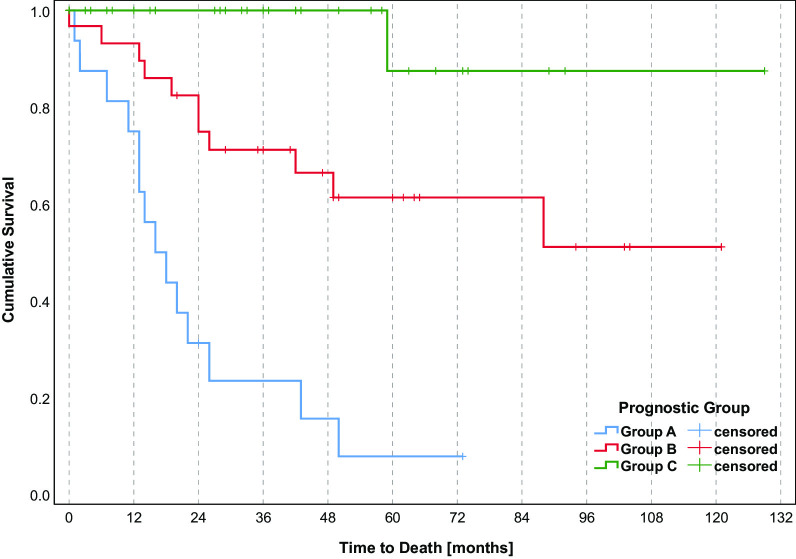
Kaplan–Meier curve for the three prognostic groups (*p* < 0.001)

Age (*p* < 0.001), KPS (*p* < 0.001), IDH status (*p* 0.006), and extent of resection (*p* < 0.001) were significantly associated with OS. Sex and MGMT promoter status failed to show significance (Table 2). On MVA, age (*p* 0.011), KPS (*p* 0.033), IDH status (*p* 0.042), and extent of resection (*p* < 0.001) all remained significant (Table 3). Kaplan–Meier curves are provided for these four characteristics that had a significant impact on OS in UVA and MVA (Fig. 1). The scoring system was based on the 36-month OS rates divided by 10. Table 4 provides an overview of the corresponding scores attributed to each characteristic. After adding the scores of the four characteristics for each patient, we obtained total scores ranging from 20 to 33 points (Fig. 2). Next, we determined three prognostic groups based on the 36-month survival rates of the patient scores: A (20–25 points), B (26–29 points), and C (30–33 points). The survival rates for the three groups were 75%, 93%, and 100% at 12 months; 23%, 71%, and 100% at 36 months; and 8%, 61%, and 88% at 60 months, respectively (Fig. 3). Median overall survival was only reached for group A at 16 months. When additionally tested for the two different primary treatment groups individually, our model score showed prognostic significance for both, the CTx only group (*p* = 0.021) and the RCTx group (*p* < 0.001).

**Table 2:** Univariate analyses

	OS rate (%)					
	12 m	24 m	36 m	48 m	*p* value	n
**Sex**					0.524	
Male	97	83	68	57		64
Female	92	70	70	70		44
**Age** **(years)**					< **0.001**	
< 41	100	91	91	85		53
≥ 41	86	58	52	43		55
**KPS** **(%)**					< **0.001**	
< 90	86	50	45	39		25
≥ 90	95	86	80	77		69
**IDH**					**0.006**	
Mutant	93	81	76	69		84
Wildtype	89	44	33	33		9
**MGMT**					0.428	
Methylated	93	75	70	66		86
Non-methylated	88	66	57	47		19
**Resection**					< **0.001**	
Biopsy	74	37	19	19		13
Subtotal	91	75	69	60		25
Gross total	97	81	80	76		65

Bold values indicate statistically significant *p* values

*OS* overall survival, *KPS* Karnofsky performance status, *IDH* isocitrate dehydrogenase type 1 and 2, *MGMT* O6-methylguanine-DNA-methyltransferase

**Table 3:** MVA for parameters significant in UVA

	HR	95% CI	*p* value
**Age** **(years)**			
< 41 versus ≥ 41	5.123	1.46–17.97	**0.011**
**KPS** **(%)**			
< 90 versus ≥ 90	0.406	0.12–0.93	**0.033**
**IDH**			
Mutant versus Wildtype	2.777	1.04–7.43	**0.042**
**Resection**			
Biopsy	Reference		<** 0.001**
Subtotal	0.157	0.05–0.52	**0.003**
Gross total	0.119	0.12–0.04	< 0.001

Bold values indicate statistically significant *p* values

*HR* hazard ratio, *CI* confidence interval, *KPS* Karnofsky performance status, *IDH* isocitrate dehydrogenase type 1 and 2

**Table 4:** Scoring points

	36 month OS rate (%)	Scoring points
**Age** **(years)**		
< 41	91	9
≥ 41	52	5
**KPS** **(%)**		
< 90	45	5
≥ 90	80	8
**IDH**		
Mutant	76	8
Wildtype	33	3
**Resection**		
Biopsy	19	2
Subtotal	69	7
Gross total	80	8

*OS* overall survival, *KPS* Karnofsky performance status, *IDH* isocitrate dehydrogenase type 1 and 2

## Discussion

We present a prognostic score designed for the primary diagnosis of AA as defined by the updated WHO classification from 2016 [1]. As no curative treatment is available, balancing a therapy with quality of life is critical in the treatment planning for patients. A survival-predicting score that is applicable at the time of primary diagnosis is a helpful tool both in expert discussions and when consulting with patients and their families. We developed a score that enables the quick discrimination of prognostic groups with significant differences in life expectancy in our cohort. A tool like this might be useful in patient consultation and when discussing aggressive versus supportive treatment approaches. The assessment of our score requires four simple variables (age, KPS, IDH status and resection status) generally available at the time of histopathological confirmation of diagnosis. No additional diagnostics are required.

The 2013 Combs score, one of the earliest prognostic scores for glioma, considers age, histology, and the time between initial RT and re-irradiation to predict survival after re-irradiation of recurrence [8]. It has repeatedly been reviewed in independent cohorts, and validation has not always been successful [15–17]. Failure to reproduce the findings established with the Combs score might partly be attributed to differences in cohort composition. For example, in 2014, Niyazi et al. attempted to reproduce the results in a patient cohort mainly treated with additional bevacizumab [18]. The 2018 re-irradiation risk score by Niyazi et al. factored in age, initial histology, and clinical performance status to predict post-recurrence survival. Their study also included an independent validation group [11]. Straube et al. recently presented a score to predict survival in elderly patients with newly diagnosed GBM, considering age, KPS, and MGMT promoter methylation [12]. As this is a GBM-specific score, it was based on a homogenous cohort of patients with GBM. All the scoring systems mentioned above were developed based on heterogeneous cohorts of patients with glioma. The proportion of patients with AA varied and was sometimes not specified. The Combs score is based on a mixed cohort of approximately 40% of patients with WHO grade II, 22% with WHO grade III, and approximately 38% with WHO grade IV tumors [8]. In the cohort investigated by Kessel et al., 64% of patients had GBM and 10% had AA (16% of WHO grade III tumors overall) [9]. In Niyazi et al.’s development cohort, 78% of patients had GBM and approximately 16% had WHO grade III tumors [11]. All authors, except for Combs et al., examined molecular parameters as potential factors in their scoring systems [8]. Kessel et al., Niyazi et al. and Straube and colleagues all considered MGMT promoter methylation status. Ultimately, it was only incorporated into the final score presented by Straube et al. Unlike our prognostic score, none of these scores include IDH status [10–12].

The importance of age at both the time of primary diagnosis and recurrence of glioma is underlined by its representation in all the mentioned prognostic scores [8, 10–12]. The role of the KPS, however, is not as unambiguous as the role of age. Although KPS was not predictive of survival after re-irradiation in the original Combs score, this finding could not be replicated by Kessel et al., who then added KPS to the prognostic score [9, 10]. When discussing the impact of resection status, careful differentiation between primary disease and recurrence is indicated. The modified Combs score incorporates whether re-resection has been performed and has shown borderline significance on MVA [10]. Niyazi et al. did not consider re-resection as a factor for score development [11]. Straube et al. considered the extent of the initial resection in UVA, but it failed to show a significant impact on MVA [12]. In our analysis, the results of UVAs and MVAs emphasize the importance of maximal safe resection as the only treatment-related prognosis-defining factor in our cohort.

In summary, the identified prognostic factors presented in our score are in line with earlier studies. A particular strength of our study is including only patients with AA, verified by tested absence of 1p/19-codeletion. Although we were able to define a score that shows significant prognostic strength, our study had several limitations.

The first limitation is the lack of molecular data. We were unable to retrieve the IDH status for about 14% of our cohort. The reason for this is that testing for a specific range of molecular parameters was not a standard diagnostic procedure only a few years prior. Partially missing information on IDH status is therefore rooted in this study’s 10-year retrospective design. However, we were still able to provide more data on molecular parameters than have been provided in comparable papers. Information on MGMT promoter methylation was fairly complete with information missing for only 3% of all patients. Ultimately, MGMT promoter methylation status did not reach statistical significance in MVA and was therefore not considered for the construction of our score. Another genetic factor not analyzed here is CDKN2A (cyclin-dependent kinase inhibitor 2A). For IDH-mutant astrocytic gliomas, homozygous deletion of the CDKN2A gene has recently been shown to be a powerful predictor of poor outcome [19]. CDKN2A should therefore be evaluated as a potential factor for use in improved future versions of the score.

The second limitation of our study is related to the rapid changes in molecular testing and classification of astrocytoma in the past few years. The current 2016 WHO classification is already considered insufficient for grading and for forming prognostic groups, and an update is expected to be released by the end of 2020 [20]. It will likely progress away from the diagnosis of IDH-wildtype astrocytoma and consider these as cases of GBM. Consequently, all diffuse astrocytomas would be IDH-mutant and future prognostic scores with the potential of implementation in clinical routine will most probably include only IDH-mutant AA.

Thirdly, postoperative treatment in our cohort is very heterogenous. This illustrates the lack of suitable AA specific treatment guidelines until recently. Several historic studies have failed to demonstrate a universally superior postoperative treatment approach [2, 4, 5, 21]. Until the CATNON trial, there was no basis for a general recommendation of combination therapy. The recent publication of the second interim analysis of the CATNON data will probably now lead to trimodal therapy being the new gold standard in treatment of IDH-mutant AA [7]. In our training cohort however, less than one fourth of the patients received trimodal therapy. We therefore consider a revised version of the score based on trimodally treated patients with IDH-mutant AA to be a reasonable next step in future research.

Fourthly, all data were acquired retrospectively and therefore were not recorded in accordance with a predefined study protocol. The KPS was not documented in some patients, and in general, the number of patients with a good KPS was relatively high in our cohort. Finally, it must be mentioned that at this point, our analysis and the score we constructed lack validation in an independent cohort.

## Conclusion

We presented a model for a score that predicts the OS of patients newly diagnosed with AA. The scoring system requires four basic characteristics that are available at the time of histopathological confirmation of diagnosis: age, KPS, IDH status and extent of resection. None of the variables require additional diagnostic workup. The retrospective design of the results presented here should be considered. A validation of the score in an independent cohort is needed. In addition, future research must be in line with our growing understanding of molecular parameters, the changing treatment approaches, and the conclusions drawn in the upcoming revision of the WHO classification. A future version meeting these criteria could serve as a simple and useful tool in the choice of treatment regimen and patient consultations.

## Data Availability

Due to data protection regulations, the data analyzed for this paper cannot be shared on a publicly available repository.

## Abbreviations

AA: Anaplastic astrocytoma
CTx: Chemotherapy
GBM: Glioblastoma
IDH: Isocitrate dehydrogenase
KPS: Karnofsky performance status
MGMT: O^6^-methylguanine-DNA-methyltransferase
MRI: Magnetic resonance imaging
MVA: Multivariable Cox regression analysis
OS: Overall survival
PFS: Progression-free survival
RCTx: Radiochemotherapy
RT: Radiotherapy
TMZ: Temozolomide
UVA: Univariate analysis
WHO: World Health Organization
